# Altered purinergic signaling in uridine adenosine tetraphosphate-induced coronary relaxation in swine with metabolic derangement

**DOI:** 10.1007/s11302-017-9563-6

**Published:** 2017-05-24

**Authors:** Zhichao Zhou, Oana Sorop, Vincent J. de Beer, Ilkka Heinonen, Caroline Cheng, A. H. Jan Danser, Dirk J. Duncker, Daphne Merkus

**Affiliations:** 1 Division of Experimental Cardiology, Department of Cardiology, Thoraxcenter, Cardiovascular Research School COEUR, Erasmus MC, University Medical Center Rotterdam, PO Box 2040, 3000 CA Rotterdam, The Netherlands; 2 Unit of Cardiology, Department of Medicine, Karolinska University Hospital, Karolinska Institutet, Stockholm, Sweden; 3 Turku PET Centre, University of Turku and Turku University Hospital, Turku, Finland; 4 Department of Clinical Physiology and Nuclear Medicine, University of Turku, Turku, Finland; 5 Department of Nephrology & Hypertension, University Medical Center Utrecht, Utrecht, The Netherlands; 6 Division of Pharmacology, Department of Internal Medicine, Erasmus MC, University Medical Center Rotterdam, Rotterdam, The Netherlands

**Keywords:** Up_4_A, Coronary relaxation, Purinergic receptor, Thromboxane, Cytochrome P_450_ 2C9, Metabolic derangement

## Abstract

We previously demonstrated that uridine adenosine tetraphosphate (Up_4_A) induces potent and partially endothelium-dependent relaxation in the healthy porcine coronary microvasculature. We subsequently showed that Up_4_A-induced porcine coronary relaxation was impaired via downregulation of P1 receptors after myocardial infarction. In view of the deleterious effect of metabolic derangement on vascular function, we hypothesized that the coronary vasodilator response to Up_4_A is impaired in metabolic derangement, and that the involvement of purinergic receptor subtypes and endothelium-derived vasoactive factors (EDVFs) is altered. Coronary small arteries, dissected from the apex of healthy swine and swine 6 months after induction of diabetes with streptozotocin and fed a high-fat diet, were mounted on wire myographs. Up_4_A (10^−9^–10^−5^ M)-induced coronary relaxation was maintained in swine with metabolic derangement compared to normal swine, despite impaired endothelium-dependent relaxation to bradykinin and despite blunted P2X_7_ receptor and NO-mediated vasodilator influences of Up_4_A. Moreover, a thromboxane-mediated vasoconstrictor influence was unmasked. In contrast, an increased Up_4_A-mediated vasodilator influence via P2Y_1_ receptors was observed, while, in response to Up_4_A, cytochrome P_450_ 2C9 switched from producing vasoconstrictor to vasodilator metabolites in swine with metabolic derangement. Coronary vascular expression of A_2A_ and P2X_7_ receptors as well as eNOS, as assessed with real-time PCR, was reduced in swine with metabolic derangement. In conclusion, although the overall coronary vasodilator response to Up_4_A was maintained in swine with metabolic derangement, the involvement of purinergic receptor subtypes and EDVF was markedly altered, revealing compensatory mechanisms among signaling pathways in Up_4_A-mediated coronary vasomotor influence in the early phase of metabolic derangement. Future studies are warranted to investigate the effects of severe metabolic derangement on coronary responses to Up_4_A.

## Introduction

Diabetes mellitus is the most prevalent endocrine disorder worldwide, and diabetes mellitus and associated metabolic derangement constitute an important risk factor for development of cardiovascular disease including atherosclerosis and diabetic heart disease. The latter is the consequence not only of proximal obstructive coronary artery disease but also of coronary microvascular disease [1, 2]. Endothelial dysfunction is an important determinant of altered vascular reactivity and plays a major role in the etiology of diabetes-induced macrovascular and microvascular complications [3, 4]. This endothelial dysfunction encompasses an imbalance between the secretion of endothelium-derived relaxing factors (such as NO and prostacyclin) and endothelium-derived constricting factors (such as endothelin and thromboxane) [4, 5].

Uridine adenosine tetraphosphate (Up_4_A) was initially identified as an endothelium-derived vasoconstrictor, exerting its constrictor influence in various vascular beds [6–10]. The vasoconstriction was shown to involve the generation of thromboxane [11] and reactive oxygen species [12]. A subsequent study reported that Up_4_A-mediated vascular contraction was enhanced in renal, basilar, and femoral arteries of DOCA-salt-induced hypertensive rats compared to normal rats [9]. Moreover, Up_4_A-induced contraction was increased in renal arteries from rats with type 2 diabetes through activation of the cyclooxygenase-thromboxane pathway [13], suggesting that disease states, including diabetes-associated metabolic derangement [14], may aggravate Up_4_A-mediated vasoconstriction.

Similar to other extracellular nucleotides, Up_4_A exerts its vasomotor influence by binding to purinergic receptors [15, 16]. The purinergic receptor family consists of P1 (adenosine receptors) and P2 receptor subtypes that can be further divided into P2X and P2Y receptors [17]. There is evidence from rodent models and humans that vascular purinergic signaling is altered in metabolic disorders [18]. The vasoconstrictor response to ATP, a putative P2 receptor agonist [19], was increased, while in preconstricted mesenteric arteries from rats with diabetes, the vasodilator response to ATP was decreased [20, 21]. Similarly, the vasodilation to ATP, UTP, and adenosine was impaired in femoral arteries of patients with type 2 diabetes [22].

Contrary to the vasoconstrictor effect of Up_4_A in most vascular beds [6–10], Up_4_A acts as a potent vasodilator in the coronary microcirculation [15]. We recently demonstrated that Up_4_A-induced coronary relaxation was blunted in swine after myocardial infarction possibly via downregulation of P1 receptors [23]. In the present study, we tested the hypothesis that the coronary vasodilator response to Up_4_A is also impaired in swine with metabolic derangement (diabetes mellitus and dyslipidemia) and investigated the involvement of altered signaling through purinergic receptor subtypes and endothelium-derived vasoactive factors (EDVFs). Our findings surprisingly demonstrate that, despite the presence of endothelial dysfunction, the coronary vasodilator response to Up_4_A was maintained in metabolic derangement. However, the contribution of purinergic receptor subtypes and endothelium-derived factors was markedly altered.

## Materials and methods

### Animals

Diabetes was induced in 10 female swine (2–3 months old, 25.1 ± 1.2 kg) with intravenous injections of streptozotocin (50 mg/kg/day, Bioconnect, The Netherlands, AG-CN2-0046) for three days. This dose of streptozotocin resulted in a stable hyperglycemia, as evidenced by glucose levels of 19.5 ± 1.4 (week 1), 21.6 ± 2.9 (week 3), 18.8 ± 2.0 (week 18), and 18.7 ± 2.1 mmol/l (week 20). One to two weeks after diabetes induction, a high-fat diet (25% saturated fats and 1% cholesterol) was gradually introduced [3]. Swine were housed in metabolic cages with ad libitum access to food for 1 h per meal, twice daily for the entire 6-month study duration. The diabetic status was regularly monitored by measurement of glucose and ketone levels in urine and blood. At sacrifice, swine with metabolic derangement weighed 104 ± 8 kg. Five healthy female crossbred Yorkshire × Landrace swine, purchased from the same breeder two weeks prior to sacrifice and matched for age and weight to swine with metabolic derangement (8–9 months old, 119 ± 5 kg), served as a control group.

### Blood and tissue sampling

At sacrifice, animals were sedated with an intramuscular injection of Zoletil (Tiletamine/Zolazepam; 5 mg/kg) and Xylazine (2.25 mg/kg), anesthetized with pentobarbital (20 mg/kg/h i.v.) and artificially ventilated. Catheters were inserted for arterial blood sampling [24]. The collected blood samples were stored for later determination of lipids, glucose and insulin levels. Following thoracotomy, hearts were arrested and immediately excised and placed in cold, oxygenated Krebs bicarbonate buffer solution.

### Myograph studies

Left ventricular apices from female swine with metabolic derangement (*n* = 10) and normal swine (*n* = 5) were collected at the time of sacrifice. Additional apices were collected from 8 hearts of healthy swine (3 female + 5 unknown gender) obtained from a local slaughterhouse. When the vascular response to Up_4_A (Biolog Life Science, Germany, U008) in the 8 female swine did not differ from the 5 swine of unknown gender from the slaughterhouse, data were pooled into a single control group (normal, *n* = 13), otherwise only data from coronary small arteries of female swine were used.

Coronary small arteries (diameter ~ 150 μm) were dissected out from the apex of 7 swine with metabolic derangement and 13 normal swine and stored overnight in cold, oxygenated Krebs bicarbonate solution of the following composition (mM): NaCl 118, KCl 4.7, CaCl_2_ 2.5, MgSO_4_ 1.2, KH_2_PO_4_ 1.2, NaHCO_3_ 25, and glucose 8.3; pH 7.4 [15, 25]. The next day, coronary arteries were cut into segments of ~2 mm length and mounted in microvascular myographs (Danish Myo Technology) in separate 6 ml organ baths containing Krebs bicarbonate solution aerated with 95% O_2_/5% CO_2_ and maintained at 37 °C. Changes in contractile force were recorded with a Harvard isometric transducer. Following a 30-min stabilization period, the internal diameter was set at a tension equivalent to 0.9 times the estimated diameter at 100 mmHg effective transmural pressure [15, 25]. At the end of the stabilization period, the vessels were exposed to 30 mM KCl twice to check the contractility. Endothelial integrity was verified by observing relaxation to 10 nM substance P after preconstriction with 100 nM of the stable thromboxane A_2_ analogue 9,11-dideoxy-11α,9α-epoxymethanoprostaglandin F2α (U46619). Then, vessels were subjected to 100 mM KCl to determine the maximal vascular contraction. Thereafter, vessels were allowed to equilibrate in fresh Krebs solution for 30 min before initiating different experimental protocols [15, 25]. In experiments where the effect of an antagonist on the response to Up_4_A was assessed, antagonists were added to the organ baths 30 min before preconstriction with U46619 and were present throughout the experiments. Only one protocol was executed per vessel segment, and, within one protocol, each vessel was obtained from a different animal.

### Experimental protocols

The coronary small arteries from both normal swine and swine with metabolic derangement were subjected to increasing Up_4_A concentrations (10^−9^–10^−5^ M). Since no vasoconstrictor influence was observed in vessels from both normal swine and swine with metabolic derangement (data not shown), vessels were preconstricted with U46619 to study the vasodilator effect of Up_4_A and the signaling pathways involved.

To investigate the involvement of purinergic receptors in Up_4_A-induced relaxation, preconstricted coronary small arteries from both normal swine and swine with metabolic derangement were exposed to increasing Up_4_A concentrations (10^−9^–10^−5^ M), in the absence and presence of the non-selective P1 receptor antagonist 8-phenyltheophylline (8PT, 10 μM, Sigma-Aldrich, The Netherlands, P2278), the non-selective P2 receptor antagonist pyridoxalphosphate-6-azophenyl-2′,4′-disulfonic acid (PPADS, 10 μM, Sigma-Aldrich, The Netherlands, P178), and combined P1 and P2 receptor blockade [15, 23]. To assess the role of purinergic receptor subtypes, vessels were tested in the absence and presence of adenosine A_2A_ receptor antagonist SCH58261 (100 nM, Sigma-Aldrich, The Netherlands, S4568), P2X_1_ receptor antagonist MRS2159 (30 μM, Sigma-Aldrich, The Netherlands, M7684) [15, 23, 26], P2X_7_ receptor antagonist A438079 (10 μM, Sigma-Aldrich, The Netherlands, A9736) [27], P2Y_1_ receptor antagonist MRS2179 (1 μM, Sigma-Aldrich, The Netherlands, M3808), or P2Y_6_ antagonist MRS2578 (10 μM, Sigma-Aldrich, The Netherlands, M0319) [15, 23]. Although the purinergic receptor blockers, in the concentrations employed in the present study, predominantly block the receptor subtypes they were designed for, it cannot be entirely excluded that other purinergic receptors were, to some extent, also affected. Careful evaluation of the selectivity profiles is therefore necessary to properly interpret the results in the present study. MRS2159 has been shown to block, in addition to P2X_1_ receptors, P2X_2_, P2X_2/3_, and P2Y_1_ receptors in 1321N1 cells [27] and P2X_7_ receptors in 1321N1 cells and erythrocytes [27, 28]. Moreover, while A438079, at concentrations up to 100 μM, has been shown to be selective for P2X_7_ receptors as compared to P2X_1_, P2X_2a_, P2X_2/3_, P2X_4_, P2Y_1_, and P2Y_2_ receptors in 1321N1 cells [29], other purinergic receptor subtypes were not tested. MRS2179 was shown to inhibit neither the response to UTP at the human P2Y_2_ and P2Y_4_ receptors nor the response to UDP at the P2Y_6_ receptor, and it was found to be 11-fold and 130-fold more selective for P2Y_1_ receptors than for P2X_1_ and P2X_3_ receptors, respectively, and to be inactive at rat P2X_2_ and P2X_4_ receptors in *Xenopus* oocytes [30]. MRS2578, at concentrations up to 10 μM, did not affect P2Y_1_, P2Y_2_, P2Y_4_, and P2Y_11_ receptor-mediated responses in 1321N1 cells [31]. Finally, SCH58261 was shown to be 581-fold more selective for A_2A_ than A_2B_ receptors [32].

A subset of coronary small arteries was de-endothelialized, preconstricted with 100 nM U46619, and exposed to increasing Up_4_A concentrations (10^−9^–10^−5^ M). Other endothelium-intact coronary small arteries from both normal swine and swine with metabolic derangement were preconstricted with U46619 and exposed to increasing Up_4_A concentrations in the absence and presence of the nitric oxide synthase (NOS) inhibitor LNAME (100 μM, Sigma-Aldrich, The Netherlands, N5751), LNAME in combination with the cyclooxygenase inhibitor indomethacin (10 μM, Sigma-Aldrich, The Netherlands, I7378) [15], or LNAME + indomethacin + the cytochrome P_450_ 2C9 (CYP 2C9) inhibitor sulfaphenazole (10 μM, Sigma-Aldrich, The Netherlands, S0758) [15, 23]. Finally, the effect of thromboxane synthase inhibitor ozagrel (10 μM, Sigma-Aldrich, The Netherlands, O1385) on the coronary vasodilator response to Up_4_A was studied in the absence and presence of LNAME.

MRS2578 and indomethacin were firstly dissolved in DMSO and further diluted in distilled water. Sulfaphenazole was dissolved in ethanol and further diluted in distilled water. Other drugs were dissolved in distilled water. PPADS and MRS2159 were protected from light.

### Quantitative real-time PCR analysis

Following dissection, endothelium-intact coronary small arteries were snap-frozen in liquid nitrogen to be used for detection of A_2A_, P2X_1_, P2X_4_, P2X_7_, P2Y_1_, P2Y_2_, P2Y_4_, and P2Y_6_ receptors mRNA. In addition, the expression of endothelial NOS (eNOS) was measured [33]. Total RNA was extracted from 5 to 7 frozen samples per group using a Qiagen RNA kit. cDNA was synthesized from 100 ng of total RNA with iScript Reverse Transcriptase (Bio-Rad). Quantitative real-time PCR (MyIQ, Bio-Rad) was performed with SYBR Green (Bio-Rad) [15]. Target gene mRNA levels were expressed relative to the housekeeping gene glyceraldehyde-3-phosphate dehydrogenase (GAPDH) as an endogenous control [34]. Primer sequences are shown in Table 1.

**Table 1:** Primer information

Genes	Sequence		Size
	Sense	Antisense	
A_2A_	5′-ATGTTGGGCTGGAATAGCTG-3′	5′-CACGGAGTTGGTGTGAGAGA-3′	426 bp
P2X_1_	5′-TTGAACCCCATTTCTTCCTG-3′	5′-AGTGCACCACACATCTGCTC-3′	248 bp
P2X_4_	5′-TGTCCCCAGGCTACAATTTC-3′	5′-GGCAGCTTTTTCTCCCTTCT-3′	373 bp
P2X_7_	5′-CTTTTGCACCTTGAGCTTCC-3′	5′-TCCATGCTAAGGGATTCTGG-3′	152 bp
P2Y_1_	5′-TTCCTGACTTGCATCAGTGC-3′	5′-CAGTGCCCGAGTAGAAGAGG-3′	157 bp
P2Y_2_	5′-GTGGCCTACAGCTTGGTCAT-3′	5′-GCGTGCGGAAGGAGTAGTAG-3′	235 bp
P2Y_4_	5′-GACTGCCGGTTTAATGAGGA-3′	5′-AGGAAAAGGACGCTGCAGTA-3′	302 bp
P2Y_6_	5′-CTGCTCTTGCCACCTGTGTA-3′	5′-AGGTTGGCGTAGAACAGGAA-3′	251 bp
eNOS	5′-CTCTCCTGTTGGCCTGACCA-3′	5′-CCGGTTACTCAGACCCAAGG-3′	151 bp
GAPDH	5′-TCGGAGTGAACGGATTTG-3′	5′-CCTGGAAGATGGTGATGG-3′	219 bp

### Data analysis and statistics

Data are presented as mean ± SEM. Statistical analysis of plasma lipids, glucose and insulin was performed using an unpaired two-tailed *t* test. Vascular relaxation responses to Up_4_A were expressed as percentage of contraction to U46619 [15]. The effects of drug treatment on Up_4_A responses were analyzed using two-way ANOVA for repeated measures. Statistical significance was accepted when *P* < 0.05 (two-tailed).

## Results

### Characteristics of diabetes and metabolic derangement

Metabolic derangement was present six months after induction of diabetes with streptozotocin and start of a high-fat diet. Plasma levels of glucose, total cholesterol, high density lipoprotein (HDL), low density lipoprotein (LDL), and triglycerides in swine with metabolic derangement were significantly elevated as compared to normal swine, while insulin levels were similar (Table 2). Fatty streaks were observed in the proximal coronary arteries, but plaque burden never amounted more than 10% of the vascular lumen; fatty streaks were not observed in the coronary microvessels.

**Table 2:** Plasma and lipid parameters of variables

	Normal (*n* = 5)	Metabolic derangement (*n* = 10)
Insulin (pmol/l)	2.50 ± 0.48	3.42 ± 0.92
Glucose (mmol/l)	5.32 ± 0.77	16.58 ± 1.07*
Cholesterol (mmol/l)	2.22 ± 0.34	16.45 ± 2.10*
HDL (mmol/l)	0.88 ± 0.11	3.74 ± 0.59*
LDL (mmol/l)	1.38 ± 0.27	14.35 ± 1.98*
Triglycerides (mmol/l)	0.15 ± 0.04	0.63 ± 0.11*

*HDL* high density lipoprotein, *LDL*, low density lipoprotein. Values are mean ± SEM; *P < 0.05 vs. normal

### Vasoactive influence of Up_4_A on coronary vasculature in swine with metabolic derangement

**Fig. 1:**
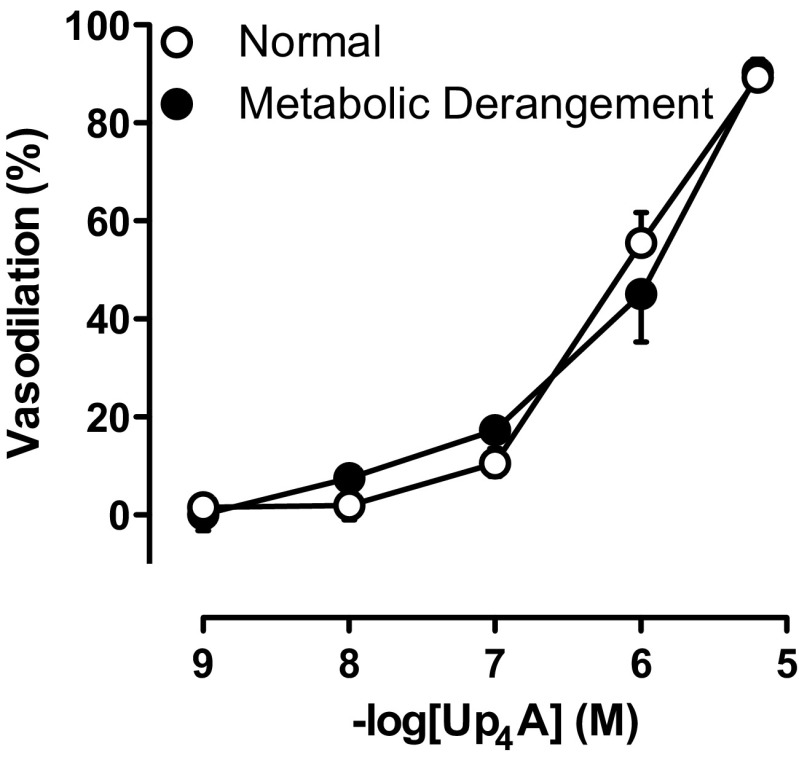
Vasodilator effects of Up_4_A. Shown are concentration responses to Up_4_A (10^−9^–10^−5^ M) in coronary small arteries preconstricted with U46619 (100 nM) from normal swine (*n* = 13) and swine with metabolic derangement (*n* = 10). Values are mean ± SEM

Up_4_A failed to elicit any vasoconstrictor response in coronary small arteries from either normal swine or swine with metabolic derangement (data not shown). Following preconstriction with U46619, Up_4_A produced concentration-dependent relaxation up to 100%, which was virtually identical between the two groups (Fig. 1). In contrast, bradykinin (10^−10^–10^−6^ M)-induced endothelium-dependent relaxation in coronary small arteries was impaired in swine with metabolic derangement (−logEC_50_ 8.61 ± 0.12 in normal (*n* = 6) vs 7.89 ± 0.19 in metabolic derangement (*n* = 5); *P* < 0.05).

### Effect of metabolic derangement on function and expression of purinergic receptor subtypes

**Fig. 2:**
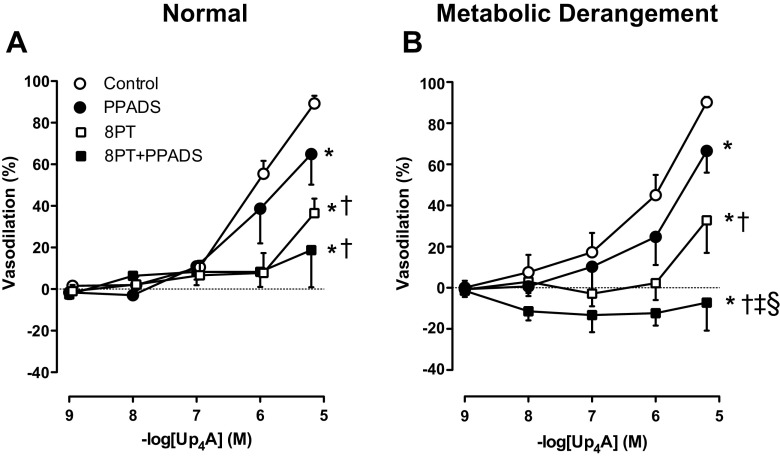
Effects of non-selective purinergic receptor blockade on Up_4_A-induced relaxation. Shown are concentration responses to Up_4_A (10^−9^–10^−5^ M) in porcine coronary small arteries in the control vessels (normal, *n* = 13; metabolic derangement, *n* = 7) and in the presence of 10 μM non-selective P1 receptor antagonist 8PT (normal, *n* = 5; metabolic derangement, *n* = 6), in the presenc e of 10 μM non-selective P2 receptor antagonist PPADS (normal, *n* = 5; metabolic derangement, *n* = 7) as well as in the presence of combined 8PT and PPADS (normal, *n* = 6; metabolic derangement, *n* = 4). Values are mean ± SEM. *P < 0.05 vs. Control; †*P* < 0.05 vs. PPADS; ‡*P* < 0.05 vs. 8PT; §*P* < 0.05 effect of 8PT + PPADS in metabolic derangement differs from that in normal

Non-selective P1 receptor blockade with 8PT and, to a lesser extent, non-selective P2 receptor blockade with PPADS attenuated the coronary vasodilator response to Up_4_A (Fig. 2). These responses were however not different between the two groups. In contrast, while the effect of combined administration of 8PT and PPADS was similar to the effect of 8PT alone in normal swine (Fig. 2a), the effect of 8PT and PPADS was significantly greater than the effect of 8PT alone in swine with metabolic derangement with unmasking of a mild vasoconstrictor response to Up_4_A (Fig. 2b).

**Fig. 3:**
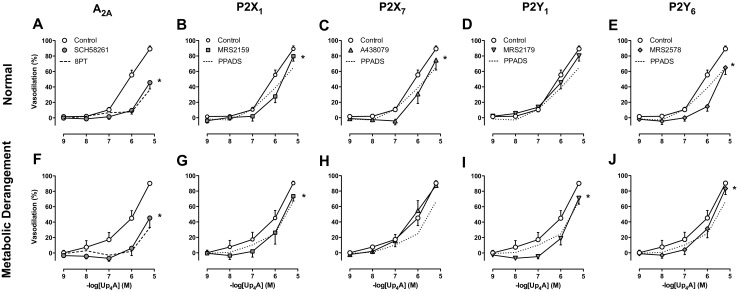
Involvement of purinergic receptor subtypes in Up_4_A-induced relaxation. Shown are concentration responses to Up_4_A (10^−9^–10^−5^ M) in porcine coronary small arteries in control vessels (normal, *n* = 13; metabolic derangement, *n* = 7) and in the presence of 100 nM A_2A_ receptor antagonist SCH58261 (normal (*n* = 8, panel **a**); metabolic derangement (*n* = 7, panel **f**)); in the presence of 30 μM P2X_1_ receptor antagonist MRS2159 (normal (*n* = 6, panel **b**); metabolic derangement (*n* = 7, panel **g**)); in the presence of 10 μM P2X_7_ receptor antagonist A438079 (normal (*n* = 7, panel **c**); metabolic derangement (*n* = 6, panel **h**)); in the presence of 1 μM P2Y_1_ receptor antagonist MRS2179 (normal (*n* = 9, panel **d**); metabolic derangement (*n* = 7, panel **i**)); and in the presence of 10 μM P2Y_6_ receptor antagonist MRS2578 (normal (*n* = 6, panel **e**); metabolic derangement (*n* = 6, panel **j**)). For the sake of comparison, non-selective P1 blockade with 8PT (normal, *n* = 10; metabolic derangement, *n* = 6) is shown as *dotted line* in panels **a** and **f**, while non-selective P2 blockade with PPADS (normal, *n* = 5; metabolic derangement, *n* = 7) is shown as *dotted line* in panels **b**, **c**, **d**, **e**, **g**, **h**, **i**, and **j**. Values are mean ± SEM. *P < 0.05 vs. control

**Fig. 4:**
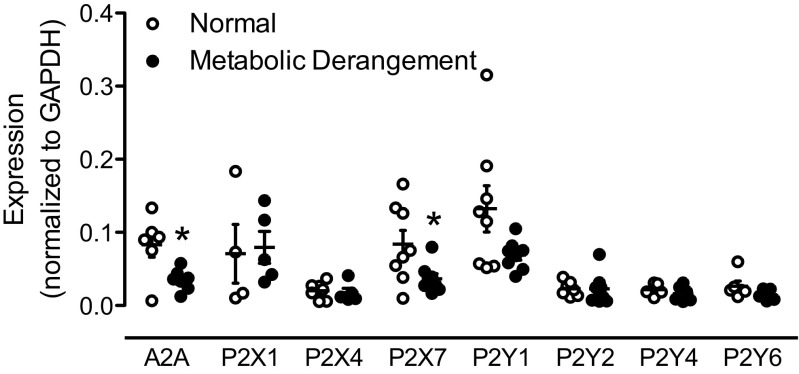
Expression profile of various purinergic receptor subtypes in normal and metabolic derangement. mRNA levels of each gene were normalized to that of housekeeping gene GAPDH. Data in the normal group are identical to those published in [23] but were performed in parallel to the experiments in the vessels obtained from animals with metabolic derangement. Values are mean ± SEM. *P < 0.05 vs. normal

A_2A_ receptor blockade resulted in attenuation of the response to Up_4_A that was comparable with the attenuation by 8PT and was not different between coronary small arteries from normal swine and swine with metabolic derangement (Fig. 3a, f), despite slightly lower A_2A_ mRNA expression levels in coronary small arteries of metabolic derangement (Fig. 4). P2X_1_ receptor blockade with MRS2159 attenuated Up_4_A-induced relaxation to a similar extent in coronary small arteries from normal swine and swine with metabolic derangement (Fig. 3b, g), which was consistent with the unaltered P2X_1_ mRNA expression between the two groups (Fig. 4). P2X_7_ receptor blockade with A438079 attenuated the vasodilator response to Up_4_A in coronary small arteries from normal swine (Fig. 3c) but not from swine with metabolic derangement (Fig. 3h), which was consistent with the reduced coronary P2X_7_ mRNA expression in swine with metabolic derangement (Fig. 4). In contrast, P2Y_1_ blockade with MRS2179 attenuated Up_4_A-induced relaxation only in coronary small arteries from swine with metabolic derangement (Fig. 3i) but not from normal swine (Fig. 3d) despite the unaltered mRNA expression of P2Y_1_ receptors (Fig. 4). The attenuation by P2Y_6_ blockade with MRS2578 of Up_4_A-induced vasorelaxation and the P2Y_6_ mRNA expression were comparable between coronary small arteries of normal swine and swine with metabolic derangement (Figs. 3e, j and 4).

### Role of endothelium in Up_4_A-induced coronary relaxation

**Fig. 5:**
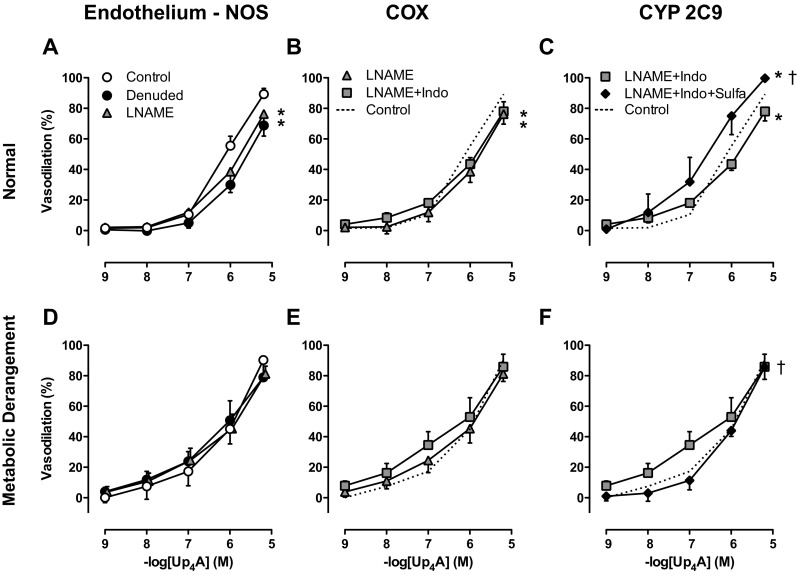
Involvement of the endothelium and endothelium-derived vasoactive factors in Up_4_A-induced relaxation. Shown are concentration responses to Up_4_A (10^−9^–10^−5^ M) in porcine coronary small arteries in the absence and presence of endothelium (normal (*n* = 13 with endothelium; *n* = 7 without endothelium, panel **a**); metabolic derangement (*n* = 7 with endothelium; *n* = 6 without endothelium, panel **d**)); in the presence of 100 μM of the NOS inhibitor LNAME (normal (*n* = 8, panel **a**); metabolic derangement (*n* = 7, panel **d**)); in the presence of combined LNAME and 10 μM of the COX inhibitor indomethacin (indo, normal (*n* = 7, panel **b**); metabolic derangement (*n* = 7, panel **e**)); and in the presence of combined LNAME, indo, and 10 μM CYP 2C9 inhibitor sulfaphenazole (sulfa, normal (*n* = 4, panel **c**); metabolic derangement (*n* = 4, panel **f**)). For the sake of comparison, the effect of Up_4_A in the absence of any blockers is shown as *dotted line* in panels **b**, **c**, **e**, and **f**. Values are mean ± SEM. *P < 0.05 vs. control; †*P* < 0.05 effect of sulfa after LNAME + indo

Endothelial denudation, as confirmed by the absence of a vasodilator response to substance P (data not shown), attenuated the vasodilator response to Up_4_A in coronary small arteries from normal swine (Fig. 5a) but not from swine with metabolic derangement (Fig. 5d). Similarly, eNOS inhibition with LNAME attenuated the response to Up_4_A in vessels from normal swine (Fig. 5a) but not from swine with metabolic derangement (Fig. 5d), which was consistent with the reduced eNOS expression in swine with metabolic derangement, from 0.52 ± 0.14 in normal to 0.19 ± 0.03 in metabolic derangement (*P* < 0.05). Additional COX-inhibition with indomethacin had no effect in coronary small arteries from either normal swine (Fig. 5b) or swine with metabolic derangement (Fig. 5e). In the presence of combined eNOS and COX blockade, inhibition of CYP 2C9 with sulfaphenazole enhanced the relaxation to Up_4_A in coronary small arteries from normal swine, (Fig. 5c), whereas in vessels from swine with metabolic derangement, sulfaphenazole attenuated the vasorelaxation to Up_4_A (Fig. 5f). These findings suggest that CYP 2C9 switches from the production of vasoconstrictor metabolites in normal swine to vasodilator metabolites in swine with metabolic derangement.

**Fig. 6:**
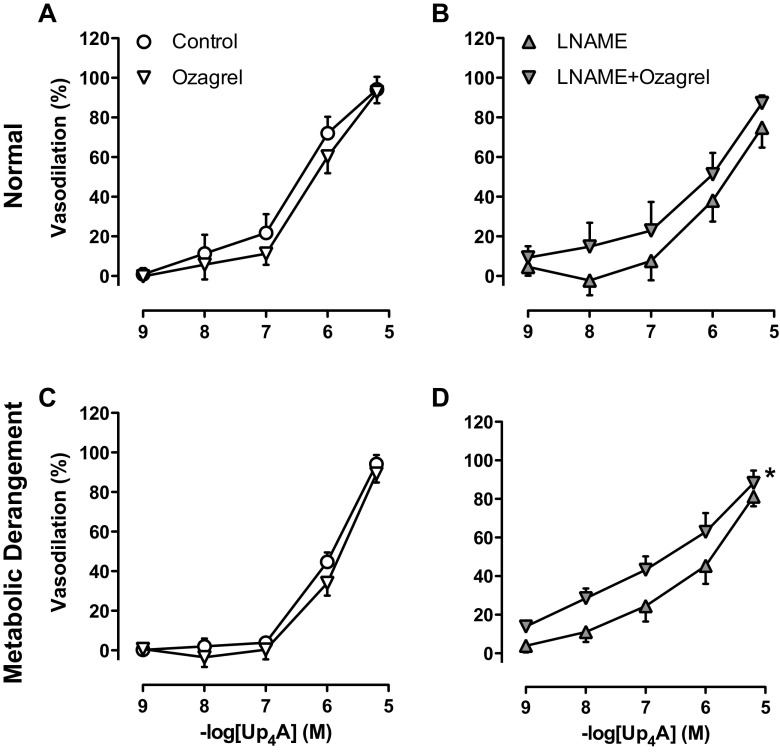
Involvement of thromboxane in Up_4_A-induced relaxation. Shown are concentration responses to Up_4_A (10^−9^–10^−5^ M) in the presence and absence of 10 μM of the thromboxane synthase inhibitor ozagrel in porcine coronary small arteries (normal (*n* = 5, panel **a**); metabolic derangement (*n* = 4, panel **c**)), as well as in the presence of 100 μM of the NOS inhibitor LNAME (normal (*n* = 6, panel **b**); metabolic derangement (*n* = 7, panel **d**)). Values are mean ± SEM. *P < 0.05 effect of ozagrel vs. corresponding control

The thromboxane synthase inhibitor ozagrel had no effect on Up_4_A-induced relaxation in coronary small arteries from either normal swine (Fig. 6a) or swine with metabolic derangement (Fig. 6c). However, while in normal swine, in the presence of LNAME, ozagrel failed to significantly affect Up_4_A-induced relaxation of coronary small arteries (Fig. 6b, *P* = 0.17), ozagrel significantly enhanced the vasodilator response to Up_4_A in coronary small arteries from swine with metabolic derangement (Fig. 6d).

## Discussion

The present study is the first to investigate the coronary vascular effects of the dinucleotide Up_4_A in a large animal model of metabolic derangement. The main findings were that (1) Up_4_A-induced relaxation was not altered in coronary small arteries isolated from swine with metabolic derangement as compared to normal swine, despite the presence of endothelial dysfunction; (2) combined P1 and P2 receptor blockade had an additive effect in swine with metabolic derangement but not in normal swine; (3) there was a shift in involvement of purinergic receptors from P2X_7_ receptors in normal swine to P2Y_1_ receptors in swine with metabolic derangement; (4) endothelial denudation as well as eNOS inhibition attenuated the response to Up_4_A in normal swine but not in swine with metabolic derangement, and eNOS inhibition unmasked thromboxane production in swine with metabolic derangement, but not normal swine, in response to Up_4_A; and (5) CYP 2C9 metabolites exerted a vasoconstrictor influence in response to Up_4_A in normal swine but exerted a vasodilator influence on the Up_4_A responses in swine with metabolic derangement. The implications of these findings will be discussed below.

The dinucleotide Up_4_A was initially identified as a novel endothelium-derived vasoconstrictor [6]. Subsequent studies demonstrated that Up_4_A induces vascular contraction in most vascular beds, including mouse renal arterioles [35], mouse aortas [11, 36, 37], rat aortas [12], rat mesenteric arteries [9], rat pulmonary arteries [7], and rat renal arteries [6], particularly in the presence of diabetes [13]. In addition to its vasoconstrictor effects in most vascular beds under basal tone, Up_4_A can act as a vasodilator when vascular tone is elevated in the rat aortas and rat perfused kidney [12, 38]. In contrast, we previously found that Up_4_A is purely a vasodilator in the healthy porcine coronary microcirculation, without any vasoconstrictor activity [15]. We also recently found that this vasodilator response to Up_4_A is reduced after myocardial infarction in coronary microvessels supplying the remote myocardium [23]. Surprisingly, we did not observe any blunting of the coronary vasodilator response to Up_4_A in swine with metabolic derangement as compared to normal swine in the present study (Fig. 1), despite evidence of endothelial dysfunction as indicated by the blunted endothelium-dependent relaxation to bradykinin both at 2 months in our previous study [3] as well as 6 months of metabolic derangement in the present study. However, we did observe marked changes in the contribution of various purinergic receptor and endothelial mediators to the relaxation produced by Up_4_A.

In coronary small arteries from healthy swine, the vasodilator effect of Up_4_A is principally mediated through P1 receptors, although the P2X_1_ and P2Y_1_ receptors also contribute in vessels of approximately 150 μm in diameter [23] but not in vessels of 250–300 μm [15]. In coronary small arteries from swine with metabolic derangement, expression of the A_2A_ receptor was slightly reduced, but a major part of the vasodilator effect of Up_4_A was still exerted through this purinergic P1 receptor subtype. The effect of combined P1 and P2 receptor blockade was markedly enhanced in the coronary small arteries from swine with metabolic derangement, even unmasking a modest vasoconstrictor effect in response to Up_4_A, suggesting that the P2 receptor subtypes involved in response to Up_4_A may differ between the two groups. Indeed, we found that both the expression and contribution of P2X_7_ receptors to Up_4_A-induced relaxation were decreased. In contrast, the contribution of P2Y_1_ receptors was increased in coronary small arteries from swine with metabolic derangement, even though expression of the P2Y_1_ receptors was not altered. The apparent discrepancy between receptor expression and function is likely due to the fact that receptor mRNA does not equate to receptor function. In addition, expression was studied in whole vessels so that we cannot distinguish expression in endothelial cells from vascular smooth muscle cells. P2 receptors in the vasculature are present on endothelial cells as well as on vascular smooth muscle cells. Activation of P2X_1_ receptors and, in some vascular beds, P2X_2_, P2X_4_ and P2Y_1_, P2Y_2_, and P2Y_6_ receptors on smooth muscle cells, generally results in vasoconstriction, although vasodilation is sometimes also observed [39]. Activation of P2Y_1_, P2Y_2_ and possibly also P2Y_4_, P2Y_11_, P2X_1_, P2X_2_, P2X_3_, P2X_4_, and P2X_7_ receptors on endothelial cells leads to the production of nitric oxide (NO) and subsequent vasodilation [39, 40]. It is therefore possible that a shift in expression of the P2Y_1_ receptors from vascular smooth muscle to endothelium may have resulted in an increased contribution of these receptors to the vasodilator effect of Up_4_A. Interestingly, with age, a shift in P2Y_1_ receptors from endothelial cells to vascular smooth muscle cells was observed in the rat cerebral vasculature [41]. Consistent with these findings, we observed an attenuation of Up_4_A-induced vasorelaxation by P2Y_1_-receptor blockade in our previous study in healthy swine of approximately 4 months of age [23] but not in the present study in healthy swine of approximately 9 months of age. These findings also suggest that metabolic derangement may have prevented this age-dependent shift from endothelial to smooth muscle cell expression of P2Y_1_. Future studies are needed to investigate the metabolic disorder-induced changes in purinergic receptor subtype distribution between coronary endothelial and vascular smooth muscle cells in more detail.

Metabolic derangement is accompanied by microvascular dysfunction and a shift in the balance between endothelium-derived vasodilators and vasoconstrictors [4, 5]. In the present study, endothelial denudation blunted Up_4_A-induced relaxation in coronary small arteries from normal swine but not swine with metabolic derangement. This effect of endothelial denudation was similar to the effect of eNOS inhibition with LNAME, indicating a reduced involvement of NO in the vasodilator response to Up_4_A in metabolic derangement. Reduced eNOS activity, which is a hallmark of coronary endothelial dysfunction in humans with metabolic derangement [42], was also observed in our previous study in coronary small arteries [3] and is consistent with the reduced eNOS expression in swine with metabolic derangement observed in the present study.

Endothelial dysfunction often results in a shift in production of endothelium-derived vasodilators, e.g., from NO to prostanoids and EDHF [43], and an increase in endothelium-derived vasoconstrictors such as thromboxane [44, 45]. In the present study, we found that inhibition of COX had no effect in coronary small arteries from either normal swine or swine with metabolic derangement while the thromboxane synthase inhibitor ozagrel significantly enhanced the vasodilator response to Up_4_A in the presence of eNOS inhibition in swine with metabolic derangement but not in normal swine, consistent with thromboxane production in response to Up_4_A. However, although NO-production was reduced in the coronary microvasculature of swine with metabolic derangement, there was no effect of thromboxane synthase inhibition in coronary small arteries with active eNOS, suggesting that even in metabolic derangement, NO suppresses the production of thromboxane. An increased contribution of thromboxane to vascular contraction in response to Up_4_A was also recently shown in renal arteries from type 2 diabetic rats, although in that study the increased contribution of thromboxane appeared to be due to an increased sensitivity rather than an increased production of thromboxane [13]. However, in the present study, constriction to the stable thromboxane mimetic U46619 was not different between normal swine and swine with metabolic derangement (data not shown), indicating that sensitivity to thromboxane was not altered. A potential mechanism that has been proposed to underlie the increased thromboxane production in metabolic derangement is that low-grade inflammation results in eNOS uncoupling, which subsequently leads to the production of reactive nitrogen species such as peroxinitrite, that is capable of inactivating PGI_2_-synthase and cause a shift in production from prostacyclin to thromboxane [44, 45].

EDHF has been demonstrated to compensate for the loss of NO in metabolic disorder [46], and CYP 2C9 has been reported to generate EETs that can act as an EDHF in the coronary vasculature [47]. In addition to these vasodilator EETs, CYP 2C9 can also produce ROS [48], which act as vasoconstrictors in the porcine coronary vasculature [49]. Although we have previously shown in pre-adolescent swine that inhibition of CYP 2C9 alone [15], or in the presence of inhibition of eNOS/COX (unpublished data), does not affect Up_4_A-induced relaxation, we found in the present study in adult swine that inhibition of CYP 2C9, in the presence of inhibition of eNOS and COX, potentiated the vasodilator response to Up_4_A in coronary small arteries from normal swine. These data are consistent with the observations that CYP 2C9 activity increases with age [50] but predominantly produces ROS in healthy porcine coronary small arteries [48]. In contrast, in coronary small arteries from swine with metabolic derangement, inhibition of CYP 2C9 in the presence of eNOS- and COX-inhibition reduced the vasorelaxation in response to Up_4_A, suggesting that CYP 2C9 switches from the production of vasoconstrictor to vasodilator metabolites in metabolic derangement. Such production of vasodilator metabolites by CYP 2C9 is consistent with other studies showing that CYP 2C9 metabolites can act as an EDHF [47] and that EDHF(s) is able to compensate for the chronic loss of NO in coronary vasculature in metabolic derangement [46].

In conclusion, while the overall vasodilator response to Up_4_A was maintained in coronary small arteries of swine with metabolic derangement, the purinergic receptor subtypes as well as the EDVFs mediating this response were markedly altered. These findings suggest the presence of compensatory mechanisms among signaling pathways in Up_4_A-mediated coronary vasomotor influences in the early phase of metabolic derangement. Future studies are warranted to investigate the effects of severe metabolic derangement on coronary responses to Up_4_A.
